# Whole consultation simulation in undergraduate surgical education: a breast clinic case study

**DOI:** 10.1186/s12909-021-02757-x

**Published:** 2021-05-28

**Authors:** Alice Lee, Dalia Abdulhussein, Mohammad Fallaha, Olivia Buckeldee, Rory Morrice, Kathleen Leedham-Green

**Affiliations:** 1 Department of Surgery and Cancer, Imperial College London, London, SW7 2AZ UK; 2 Faculty of Medicine, Imperial College London, London, SW7 2AZ UK; 3 Medical Education Research Unit, Faculty of Medicine, Imperial College London, London, SW7 2AZ UK

**Keywords:** Simulation, Undergraduate, Medical education, Surgery, Breast, SECO

## Abstract

**Background:**

Safe and effective clinical outcomes (SECO) clinics enable medical students to integrate clinical knowledge and skills within simulated environments. This realistic format may better prepare students for clinical practice. We aimed to evaluate how simulated surgical clinics based on the SECO framework aligned with students’ educational priorities in comparison with didactic tutorials.

**Methods:**

We delivered two breast surgery SECO-based simulated clinics to Year 3 students during their surgical attachments at a London teaching hospital. All students attended a didactic breast surgery tutorial the previous week. Pre- and post-session surveys and post-session debriefs were used to explore learning gain, processes, preferences and impacts on motivation to learn. Data were analysed using inductive thematic analysis to categorise student views into themes.

**Results:**

Seventeen students enrolled in the simulated clinics and debriefs. Students expressed that passing examinations was a key extrinsic motivating factor, although the SECO-based format appeared to shift their motivation for learning towards aspiring to be clinically competent. Self-reported confidence in clinical skills such as history taking and examination improved significantly. Active learning methods were valued. Students expressed a preference for simulated clinics to complement, but not replace, tutorial-based learning.

**Conclusion:**

The SECO-based simulated clinic promoted a shift towards intrinsic motivation for learning by allowing students to recognise the importance of preparing for clinical practice in addition to passing examinations. Integration of surgical simulated clinics into the undergraduate curriculum could facilitate acquisition of clinical skills through active learning, a method highly valued by students.

**Supplementary Information:**

The online version contains supplementary material available at 10.1186/s12909-021-02757-x.

## Introduction

In the United Kingdom, the General Medical Council (GMC) sets out ‘outcomes for graduates’ which stipulate that medical graduates must show competency in safely diagnosing, investigating and managing clinical presentations across both community and secondary care settings, including surgery [1]. Elsewhere, approximately 30% of the clinical knowledge component of the United States Medical Licencing Exam is surgical, reflecting the importance of surgical knowledge in medical graduates [2]. Most newly qualified doctors rotate through surgical specialties [3] making preparedness for practice a key outcome for undergraduate surgical education.

Despite significant variation in undergraduate curricular design and recent innovations to improve practical clinical learning, a large proportion of newly qualified doctors continue to feel unprepared when transitioning to clinical practice [4–7]. Unpreparedness is related to a lack of meaningful clinical experience as an undergraduate [8]. In surgery, this is exacerbated by increasing curricular emphasis on generalism and community-based teaching which has reduced exposure [9]. Newly graduating doctors also describe a lack of clinical responsibility during their undergraduate years, affording them limited opportunities to develop skills in clinical diagnosis or the management of patients’ problems [10]. Undergraduate surgical education typically involves core knowledge in anatomy, physiology and pathology, supplemented by clinical attachments and clinical skills training. There are calls for medical educators to create more integrated learning experiences rather than separating clinical knowledge, skills, professionalism and communication [11]. Surgical placements, although highly influential in modulating career intentions in surgery [12], have multiple barriers which limit their capacity and effectiveness as an undergraduate learning environment [9, 13]. There is a pressing need for innovative ways to support students in experiential surgical learning: a central component in the integration of knowledge and skills, and the journey towards preparedness for practice [14–16]. Simulation-based training can provide integrated learning experiences and is promoted for junior surgical and medical trainees in the UK [17, 18], but is not commonplace in undergraduate training.

## Methods

### Design

In this evaluative case study, we present our teaching materials and processes, alongside a qualitative analysis of the simulation debrief which explored participants sense-making about their learning preferences and motivations. Quantitative data were collected through pre- and post-teaching confidence scales to assess factual, procedural and conceptual knowledge gains.

### The intervention

Whole consultation simulations that emphasise patient-centred outcomes within authentic clinical contexts and resources were pioneered by Williamson et al. as Safe and Effective Clinical Outcomes (SECO) clinics. These multi-part simulations require the learner, in the role of the simulated doctor to take a history from a simulated patient (actor), examine them (if appropriate), request and interpret appropriate investigations and suggest an appropriate management plan. SECO clinics address learning objectives that are clearly aligned with future clinical practice in a safe and supervised environment [19, 20]. Through this framework, students are encouraged to take clinical responsibility for patients by combining their knowledge and clinical reasoning skills to make decisions, including seeking appropriate advice in order to achieve safe, effective outcomes. SECO clinics have been evaluated as both engaging and effective for learning around patient-centred clinical practice in primary care [20, 21], but have yet to be evaluated in surgical education.

**Fig. 1:**
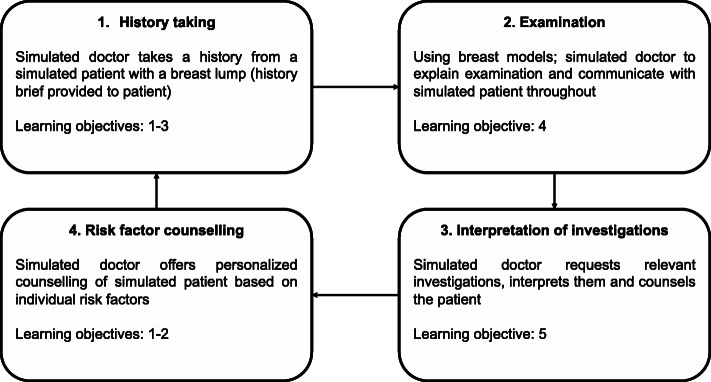
Format of the breast surgery clinic session with learning objectives (numbered in Table 1) mapped to each station

We based our simulated clinic on the SECO design [20] with pragmatic modifications. We divided the students into pairs or trios. Student groups rotated through four simulated consultations over approximately an hour (Fig. 1). Students alternated between the roles of a simulated patient and doctor, whilst being observed by a tutor (AL, DA, MF). We designed the session to feel as realistic as possible, with the provision of the usual resources available to doctors in clinics such as clerking proformas, investigation reports and management guidelines. We encouraged students to discuss any uncertainties with a ‘senior’ (role played by the tutor) as would be expected in clinical practice. We encouraged students to remain in role as a doctor throughout the simulated clinical encounter to promote awareness of their professional boundaries and capabilities.

We chose a ‘triple assessment’ breast cancer clinic for our pilot as this requires students to demonstrate a wide range of patient-centred skills. Triple assessment clinics involve taking a focussed history, performing an examination and selecting the correct choice of imaging and histology. The examination was performed on a standard, wearable breast model (‘Breast Examination Trainers’, available from *Limbs & Things©*) worn by another student, with the option of inserting lump “pathology” which can be palpated by students on examination. All students were presented with the same finding of a solitary lump detected on breast examination. Students were required to order the correct tests based on their history and examination, interpret the results, and communicate their findings and plan. Participants had all participated in an hour-long classroom-based breast tutorial, facilitated by OB, the previous week. The learning outcomes were taken from the year 3 curriculum at Imperial College London (Table 1).

**Table 1:** Learning outcomes for the Virtual Breast clinic (based on the Imperial College London curriculum)

**Learning outcomes**	
1. Explain the aetiology/risk factors for breast cancer 2. Summarise the epidemiology of breast cancer 3. Recognise the presenting symptoms of breast cancer 4. Recognise the signs of breast cancer on physical examination 5. Identify appropriate investigations for breast cancer and interpret the results	

We provided feedback to students in two formats (as per the original SECO design) per station: i) achievement of clinical outcomes and ii) achievement of patient-centred outcomes, assessed by the tutor and simulated patient, respectively. Examples of station materials and feedback forms can be found in the supplementary material.

### Research aims

Our primary aim was to explore and categorise factors influencing medical students’ motivation to learn, and how these were modulated by their experiences during the simulated clinic. Our secondary aims were to explore students’ perceptions of simulated clinics in comparison to classroom-based surgical education to help characterise their preferences for learning, and to identify skill and knowledge domains where learner confidence increased, to characterise knowledge gain.

### Research team

OB and RM are hospital-based clinical teaching fellows, whilst AL, MF and DA are hospital-based junior doctors. KLG is a full-time medical education researcher. OB, RM and KLG have post-graduate qualifications in education and designed the project.

### Participants and setting

Students (*n* = 17) were in their third year of a six-year medical degree. This was their first year of clinical placements and their first general surgical placement. We delivered these sessions at a North West London teaching hospital in November 2019 and January 2020 with eight students in the first session and nine students in the second session. All students were invited to participate and 17 gave written consent for their data to be used for evaluative purposes.

### Data generation

Immediately after the session, students participated in a 20–30 min debrief. This was recorded with informed written consent. OB and RM led the debrief sessions which followed a semi-structured format of open-ended questions, exploring experiences, preferences and impacts on motivation to learn. The topic guide was designed by OB and RM and informed by the literature on SECO clinics. We collected quantitative feedback using pre- and post-session online questionnaires. Students were asked to grade their confidence performing various clinical skills relevant to breast surgery using 5-point Likert scales (ranging from strongly disagree to strongly agree). The questionnaire and topic guide are shown in Table 2.

**Table 2:** Statements and questions used in the pre- and post- session online survey and debrief sessions. The survey used a 5-point Likert scale (from strongly disagree to strongly agree)

Likert-scale questionnaire	Debrief sessions
1. I feel confident taking a history of a patient with a breast lump 2. I feel confident documenting a history of a patient with a breast lump in clinical notes 3. I know the risk factors for breast cancer and how to ask these in a history 4. I know the presenting symptoms of breast cancer and how to ask these in a history 5. I feel confident examining a patient with a breast lump and documenting this in the clinical notes 6. I know how to recognise the examination findings of breast cancer exam 7. I would feel confident ordering appropriate investigations for a patient presenting with a breast lump 8. I would feel confident interpreting appropriate investigations for a patient presenting with a breast lump	1. How did it feel to learn in a simulated clinic environment? 2. The simulated clinic was based on the same learning outcomes as the breast tutorial last week. How did you feel that each session addressed these outcomes? 3. Do you prefer to learn in a simulated clinic environment or a classroom-based tutorial on the same subject matter? 4. Students are presented with two statements: *“The top priority in my education should be to prepare me to pass my final exams. The top priority in my education should be to prepare me to become a competent junior doctor”.* Discuss to what extent you agree or disagree with those statements and why. 5. How do you feel simulated clinic sessions and classroom-based tutorials prepare you for these educational priorities (passing examinations vs. junior doctor competencies)?

### Data analysis

The post-session debrief was transcribed verbatim and anonymised prior to analysis. We used an inductive thematic approach [22] facilitated by Dedoose online software. AL, DA and MF independently coded the debrief transcripts line-by-line with the generation of themes and sub-themes through a collaborative iterative process, the methodology of which has been described in the literature previously [22]. This involved merging duplicate codes and themes through group consensus and resolving any differences in interpretation through discussion. We then further refined these themes and sub-themes into broad categories relating to impacts on learner motivation, insights into learning processes, and comparisons between classroom-based and simulation-based learning. Pre- and post- session confidence ratings from the online Likert scale questionnaire were compared using Fishers exact test (Microsoft Excel [V16.32])**.** A *P*-value of < 0.05 was considered statistically significant.

## Results and interpretation

**Fig. 2:**
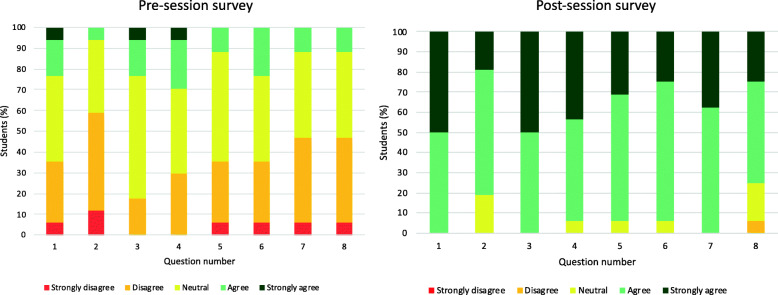
Pre- and post-teaching survey responses. Numbered questions refer to those in Table 1

For the first session (November 2019), 8 students completed the pre-course questionnaire and 7 students completed the post-course questionnaire. For the second session (January 2020), 9 students completed both the pre- and post-course questionnaire. Students reported significant improvements in their confidence across all domains. Students were initially least confident in their confidence in documenting the history and examination of a patient with a breast lump and were most confident in their ability to take a focused history from patients, and to determine symptoms and signs of breast cancer on history-taking and clinical examination respectively. The largest improvement in confidence scores was seen in the following domains: asking patients about their risk factors for breast cancer, confidence in performing a breast exam and documenting this in the notes, and confidence in ordering the correct investigations in the triple-assessment clinic (all *P* < 0.0001; Fig. 2).

For the debriefs, 17 students participated and consented to their recorded comments being used for evaluative purposes. Two debriefs ran simultaneously for each session allowing for smaller group sizes; therefore a total of four debriefs were recorded. Eight themes were identified from the sessions and these were broadly categorised into two subgroups: motivation for learning, and preferences for learning (Table 3). We do, however, acknowledge that overlap exists between these two groups.

**Table 3:** Student motivation and preferences for learning. The first four themes relate to student motivations for learning and the latter four themes relate to student preferences for learning

Theme	Sub-theme	Code(s)	Example Excerpts
Fear of failure		Avoidance of failure Avoiding mistakes on placement	Ex. 1: “I felt more confident to ask questions and get things wrong because obviously it wasn’t a real patient [...] this is the place to make mistakes rather than on the wards” Ex. 2: “But then if you do get it wrong in this [“simulated clinic”] setting then you’ve got a smaller group where you’re not disturbing too many people if you get it wrong”
Becoming clinically competent	Practical skills	Becoming competent at practical skills Practicing practical skills Understanding / practicing practical aspects of clinical practice	Ex. 3: “The breast clinic session is useful because we need to know the practical elements.” Ex. 4: “They [“simulated clinic tutors”] also made us do stuff like examinations too. For me this helps me remember stuff a bit more.”
	Communication & professionalism	Practicing communication skills Developing professional skills Knowing when to escalate and ask for help Generating good habits Temporal changes in motivation	Ex. 5: “A lot of it is skills based like being able to speak to your patient properly and then understand what they’re saying.” Ex. 6: “Just knowing the principles of history taking doesn’t actually help you that much when you come to taking a history because if you can’t make a patient feel comfortable, they won’t open up to you or talk to you.” Ex. 7: “The point of the session as well was when to ask for help and like how to do that. [...] There’s no other way really to learn about it in a textbook.” Ex. 8: “This [simulated clinic] builds your practical skills a lot better, and I feel like it gives you better information for like the history taking part.” Ex. 9: “I feel like when you get to sixth year [...] finals are approaching but then you’re also like oh a couple months after that I’m going to be the F1 and I’m going to be doing nights and covering all the wards. [...] It’s different pressures at different stages.”
	Application of clinical knowledge	Clinical reasoning Real-life practices vs textbook Having confidence in applying clinical knowledge^a^	Ex. 10: “In textbooks [...] they’ll have like a billion investigations, so you don’t necessarily know which one is the one that you’ll use first in the hospital. Whereas by doing simulated clinics you’ll see [...] this is the first line, this is what you progress to because its got better specificity.” Ex. 11: “It’s one thing to know what the symptoms are supposed to be and another thing to recognise them on a patient, even a simulated one.” Ex. 12: “Learning things like differentials, you probably get more of that in the tutorial but recognising them is an entirely different scenario.”
Acquisition of core medical knowledge		Covering the medical curriculum Acquisition of medical knowledge Learning facts	Ex. 13: “The history taking part [of the simulated clinic session] was amazing, like I personally thought that the history taking part was so useful, but actually knowing specifically about the disease and all the different investigations and symptoms and everything that can come with it can only really be well taught in a classroom environment.” Ex. 14: “We got roughly the same information out of each session (tutorials and clinic) but it was just a different way of doing it.” Ex. 15: “I wouldn’t teach about disease this [simulated clinic] way but as far as examination goes it probably makes sense to teach it this way.”
Passing examinations^b^		Passing examinations Passing non-written exams Passing written exams Temporal changes in motivation^a^	Ex. 16: “The tutorials are more like more for exams and the tutorials are more for OSCES” Ex. 17: “I think simulated clinic sessions make use a lot more competent as a junior doctor, rather than focus on helping us pass exams.” Ex. 18: “To be honest prioritising my exams is probably my priority at the moment.” Ex. 19: “The simulated sessions are useful for both competencies as a doctor as well as practical things like passing our OSCE.” Ex. 20: “If all our teaching was done in a simulated environment then I don’t think we’d feel as prepared to pass our exams.” Ex. 21: “I think at this stage, our main priority if we’re being realistic about it is to pass our exams. We can be the most competitive junior doctor, but if we haven’t passed our exams, then you know...”
Active learning techniques	Maintaining active interest during teaching exercise	Interactive teaching Active discussion Active recall / learning Assessment throughout teaching / factual recall Interactivity of the teaching session Engagement with teaching exercise	Ex. 22: “In a big group you’re less likely to put your hand up to answer a question.” Ex. 23: “It’s good to be put on the spot as well, because I think just a tutorial is quite passive and so you could be like ‘I could do that, I can do all these things’ but then when you actually go to do it in a simulated environment you’re like ‘oh wait hang on a minute.’” Ex. 24: “I think having a tutorial like a week ago not knowing what the topic is today, is probably the best thing because you learn some stuff in the tutorial, you forget about it, and then you have to use active recall to remember the info.” Ex. 25: “I learn more from practical sessions. In lunchtime tutorials there is just a lot of information so sometimes it just feels too much and it is hard to remember stuff until you actually put it to use and do it.”
	Aiding long-term memory recall	Consolidation of knowledge Forming long-lasting learning memories Memorable teaching frameworks Application of knowledge Feeling prepared for the teaching session	Ex. 26: “I think having a tutorial like a week ago not knowing what the topic is today, is probably the best thing because you learn some stuff in the tutorial, you forget about it, and then you have to use active recall to remember the info.” Ex. 27: “We already had that [tutorial] session then afterwards we came and consolidated that session a few days later with this [simulated clinic].” Ex. 28: “I think they [tutorial and simulated clinic] were both very good because i feel like the first one was more like our actual learning outcomes like our conditions that we need to know and to have a clear image of what the differentials could be and then the second one was like how would you use all this knowledge in practice.”
Teaching environment		Safety of learning environment Small group learning Suitability of learning technique Realism of clinical setting Being able to ask questions Having confidence in applying clinical knowledge^a^ Learning through mistakes^a^	Ex. 29: “I feel more at ease [...] in this environment than doing it on the ward.” Ex. 30: “I think the simulated clinic, it’s pretty much what they do in the breast clinic, so its like very much what we’ll have to do as a doctor, so I feel like in terms of that respect, this is more useful than a normal tutorial.” Ex. 31: “I felt like both [the tutorial and the simulated clinic] were useful in their own way, and in fact I felt doing this after the tutorial was actually better because it consolidated all of the stuff that we did in the tutorial so I think they both kind of go hand in hand which is kind of a good thing, but maybe just look at it as an adjuvant rather than one or the other.”
Learning through simulation		Exposure to rare learning experiences Simulated learning environment Simulation of real-life scenario Learning through mistakes^a^ Recognising clinical presentations Pure enjoyment / interactivity of the session Working on weaknesses^a^ Human factors	Ex. 32: “I don’t think well have the chance to sit in on all the clinics so this would be the place to do it to learn about things that we haven’t been able to see.” Ex. 33: “I guess it’s also [...] more time for us to go through it because it’s very rare that you see a patient from presentation to future investigations and maybe we wouldn’t have had the chance to do that on the ward.” Ex. 34: “I feel like they are making us do the stuff, like it identifies what we actually do know and what we’ve retained and what we don’t.” Ex. 35: “I feel it kind of does prepare us for the exams, but also kind of [sic] tests our professionalism and maturity.” Ex. 36: “[...] understanding your patient manner as well, and with sensitive things like breast cancer to learn how you’d approach it because you have to be more sensitive.”
Supervised and feedback-driven learning event		Feedback-led session Having time and exposure to learning experiences Quality of tutors Supervision during learning events Identify weaknesses Working on weaknesses	Ex. 37: “I think because we’re in small groups in this session it makes it easier to get quick feedback compared to in a larger tutorial.” Ex. 38: “I guess it’s also [...] more time for us to go through it because its very rare that you see a patient from presentation to future investigations and maybe we wouldn’t have had the chance to do that on the ward.” Ex. 39: “It’s more intimate, you can talk to people better and voice your concerns.” Ex. 40: “I feel like they are making us do the stuff, like it identifies what we actually do know and what we’ve retained and what we don’t.”
		Integration of SECO with existing experiences	Ex. 41: “I think having a tutorial is a good pre-session for this [simulated Clinic].” Ex. 42: “I think having a tutorial like a week ago not knowing what the topic is today, is probably the best thing because you learn some stuff in the tutorial, you forget about it, and then you have to use active recall to remember the info.” Ex. 43: “I think we would definitely still want some tutorials, like a balance is useful rather than all of one.” Ex. 44: “I would want both, but if I could only have one I’d select this because the book stuff you can just look it up on your own time whereas you can’t recreate this by yourself.” Ex. 45: “A nice idea would be to have two sessions a week, the first as classroom based and then later on that week would be simulated clinical environment to consolidate that.”

Most excerpts were tagged with multiple codes, and where it was deemed pertinent, some excerpts have been used as example excerpts more than once

^a^ Denotes code that was felt to be part of multiple themes

^b^ The authors acknowledge that examinations also act as a fear

### Impact on motivation for learning

Student motivations for engaging in learning were classified into four broad themes; fear of failure, becoming clinically competent, acquisition of core medical knowledge and passing examinations (Table 3).

Some students prioritised the acquisition of core, examinable medical knowledge, which they saw as a crucial step in passing their examinations.

Excerpt 14: “We got roughly the same information out of each session (tutorials and clinic) but it was just a different way of doing it.” Excerpt 18: “To be honest prioritising my exams is probably my priority at the moment.”

This was attributed to their stage in training. They felt that preparedness for practice would become a greater priority as they progressed through medical school.

Ex. 21: “I think at this stage, our main priority if we’re being realistic about it is to pass our exams. We can be the most competitive [sic] junior doctor, but if we haven’t passed our exams, then you know...”

Others discussed the importance of balancing codifiable knowledge and facts with more applied communication and clinical skills.

Ex. 6: “Just knowing the principles of history taking doesn’t actually help you that much when you come to taking a history because if you can’t make a patient feel comfortable, they won’t open up to you or talk to you.”

Ex. 11: “It’s one thing to know what the symptoms are supposed to be and another thing to recognise them on a patient, even a simulated one.”

Some felt the simulated clinic had reframed their academic motivation away from passing examinations and towards preparedness for practice.

Ex. 17: “I think simulated clinic sessions make us a lot more competent as a junior doctor, rather than focus on helping us pass exams.”

### Impact on student preferences for learning

Students sense-making on the processes of learning were classified into four themes; active learning techniques, teaching environment, learning through simulation, and supervised and feedback-driven learning events (Table 3).

Students welcomed the opportunity to apply their knowledge during the simulated clinic and felt that this aided their overall development of clinical competence. Active recall was valued, and this was seen by students as aiding them in consolidating their knowledge and preventing passive engagement in learning activities.

Ex. 23: “It’s good to be put on the spot as well, because I think just a tutorial is quite passive and so you could be like ‘I could do that, I can do all these things’ but then when you actually go to do it in a simulated environment you’re like ‘oh wait hang on a minute.’”

Students felt the simulated learning environment helped them to feel more comfortable trying new approaches, making mistakes, and asking questions. This enabled them to identify gaps in their learning. Students felt classroom-based learning and real surgical learning promoted passive engagement and a fear of error, whereas the simulated clinics embraced errors as an essential part of the learning process.

Ex. 1: “I felt more confident to ask questions and get things wrong because obviously it wasn’t a real patient [...] this is the place to make mistakes rather than on the wards”.

Ex. 22: “In a big group you’re less likely to put your hand up to answer a question.”

Ex. 40: “I feel like they are making us do the stuff, like it identifies what we actually do know and what we’ve retained and what we don’t.”

Comparisons were drawn between the simulated clinic and the tutorial they received on the same topic a week prior to the clinic (Table 4). Students felt the simulated clinics provided a good environment to practice practical skills but lacked the structure required to acquire more in-depth medical content. Students remarked that they felt that they would be less prepared for written exams if they only learned through simulation. They still wanted tutorials to cover the “specifics of each disease” and to ensure that “baseline knowledge” had been fully covered.

Ex. 13: “The history taking part [of the simulated clinic session] was amazing, like I personally thought that the history taking part was so useful, but actually knowing specifically about the disease and all the different investigations and symptoms and everything that can come with it can only really be well taught in a classroom environment.”

**Table 4:** Advantages and disadvantages of traditional didactic learning compared with simulated clinic learning identified by the medical students

Traditional Didactic Learning			Simulated clinic learning		
Theme	Code	Example Excerpt(s)	Theme	Code	Example Excerpt(s)
Acquisition of core medical knowledge	Covering the medical curriculum Acquisition of medical knowledge Learning facts	Ex. 13: “The history taking part [of the simulated clinic session] was amazing, like I personally thought that the history taking part was so useful, but actually knowing specifically about the disease and all the different investigations and symptoms and everything that can come with it can only really be well taught in a classroom environment.” Ex. 46: “Learning presentations and risk factors and baseline knowledge is better in a classroom.” Ex. 47: “In terms of learning things like risk factors I think the first session where there are slides is more useful as we get to see stuff.”	Passing examinations	Passing non-written exams	Ex. 19: “The simulated sessions are useful for both competencies as a doctor as well as practical things like passing our OSCE.”
Passing examinations	Passing written exams	Ex. 20: “If all our teaching was done in a simulated environment then I don’t think we’d feel as prepared to pass our exams.”	Being a competent doctor	Practical skills	Ex. 3: “The breast clinic session is useful because we need to know the practical elements.”
				Communication & professionalism	Ex. 5: “A lot of it is skills based like being able to speak to your patient properly and then understand what they’re saying.”
				Application of clinical knowledge	Ex. 10: “In textbooks [...] they’ll have like a billion investigations, so you don’t necessarily know which one is the one that you’ll use first in the hospital. Whereas by doing simulated clinics you’ll see [...] this is the first line, this is what you progress to because its got better specificity.” Ex. 12: “Learning things like differentials, you probably get more of that in the tutorial but recognising them is an entirely different scenario.”
			Active learning techniques	Maintaining active interest during teaching exercise	Ex. 22: “In a big group you’re less likely to put your hand up to answer a question.” Ex. 23: “It’s good to be put on the spot as well, because I think just a tutorial is quite passive and so you could be like ‘I could do that, I can do all these things’ but then when you actually go to do it in a simulated environment you’re like ‘oh wait hang on a minute.’”
			Learning through simulation	Simulation of real-life scenario	Ex. 30: “I think the simulated clinic, it’s pretty much what they do in the breast clinic, so its like very much what we’ll have to do as a doctor, so I feel like in terms of that respect, this is more useful than a normal tutorial.”
				Working on weaknesses	Ex. 40: “I feel like they are making us do the stuff, like it identifies what we actually do know and what we’ve retained and what we don’t.”
				Feedback-led session	Ex. 37: “I think because we’re in small groups in this session it makes it easier to get quick feedback compared to in a larger tutorial.”

### Integration of SECO with existing learning experiences

Students’ evaluations of the simulated clinic format were overall extremely positive, and many recognised the uniqueness of simulated learning events, which are a rare opportunity in their curriculum.

Ex. 44: “I would want both, but if I could only have one I’d select this [the simulated clinic] because the book stuff you can just look it up on your own time whereas you can’t recreate this by yourself.”

They did however feel that simulated clinics may not work as the sole form of delivering their surgical curriculum, but rather it should supplement their existing teaching to consolidate classroom-based learning.

Ex. 20: “If all our teaching was done in a simulated environment then I don’t think we’d feel as prepared to pass our exams.”

Ex. 45: “A nice idea would be to have two sessions a week, the first as classroom based and then later on that week would be simulated clinical environment to consolidate that.”

## Discussion

Our study identified, through inductive thematic analysis, four key themes related to student motivations for learning. These consisted of fear of failure, the need to pass examinations, the desire to acquire core medical knowledge and to become clinically competent. Furthermore, four key themes relating to student reflections on the learning preferences were identified including; active learning techniques, learning through simulation, learning through feedback and appropriateness of teaching environments. Students felt that simulated surgical clinics would be a welcome addition to the surgical curriculum, allowing practical application and consolidation of knowledge. However, they suggested that simulated clinics should not replace classroom-based teaching, which they felt was more appropriate for the delivery of high-volume semantic knowledge. In a discrete choice situation, they felt the simulated clinics were more valuable as this type of learning was not possible through self-study.

The themes surrounding learner motivation and preferences presented in this study align with previously published characteristics of adult learning in undergraduate surgical curricula [23]. These characteristics include the need for learning to be perceived as relevant, experiential, participatory, problem-based, applicable to practice and based on active, high quality feedback [23]. The requirement for learners to be included in needs assessments relating to medical training is therefore important for two reasons: to increase the perceived relevance and participatory nature of teaching and to realign the significant disparity in perceived learning needs that can exist between teachers and learners [24].

### Motivation to learn

Research has shown that engagement in educational processes is strongly linked to learner motivation [25]. Motivation can be categorized according to self-determination theory as either intrinsic or extrinsic [26]. Intrinsic motivation is driven by inherent enjoyment and satisfaction in the task, whereas extrinsic motivation usually involves external or introjected regulatory factors [27]. Examples of extrinsic motivators identified in this study include the need to pass examinations and fear of failure at work or on placement. Concern about examination performance has previously been identified as a strong motivator for learning [28]. Enjoyment of the session and interest in medicine was identified by the students in our study as an intrinsic motivator, in accordance with previous research [28].

Cook and Artino proposed five contemporary theories for motivations to learn in medical education, including self-determination, goal orientation, social-cognitive, attribution and expectancy-value [29]. Goal orientation was an important motivator in this study, with students highlighting the desire to become clinically competent, knowledgeable and adept at practical skills, both in the context of performing well as junior doctors (performance approach goal) and avoiding mistakes in examinations and on placement (performance avoidance goal). Social-cognitive theory describes reciprocal interactions between learners and their environment. In this study, students consistently praised the ability to learn through simulation, which provided an opportunity to communicate with mock patients in a realistic setting, observe their peers’ performance, and practice ‘soft skills’ such as asking for senior advice. Students placed higher task value on the simulated clinics after having had a preparatory didactic session the week before, which made them feel more confident that they could achieve their learning goals (expectancy-value theory).

During the debrief sessions, the students’ responses suggested that the simulated clinics impacted their motivation, with a shift towards intrinsic motivation. Simulation-based teaching has been reported to improve intrinsic motivation in medical students [30, 31]. Whilst passing examinations was still highly prioritized, students had a greater consideration that passing medical school examinations was only one aspect of becoming a clinically competent doctor. Use of techniques to drive intrinsic motivation is important, having been associated with improved learning outcomes, quality of care, doctor-patient relationships and reduced physician burnout and job dissatisfaction [28, 32, 33].

Internal motivation has been identified previously as a key factor associated with improved learner performance, and the whole-consultation model employed in our study also provides learners with an opportunity to be provided with well-defined goals, receive thorough feedback, and opportunities for repetition and refinement of their clinical skills and knowledge [34]. These factors are necessary for improving performance and have collectively been referred to as “deliberate practice” [34]; simulated clinics provide teachers and learners an ideal opportunity to utilise this concept to improve learner performance.

### Learning preferences

The students’ preference for active learning in a small-group simulated environment demonstrated in our study is supported in the literature and has been shown to improve skill acquisition in comparison to traditional clinical education [35–37]. Previous research has also shown that medical students value the ability to learn through realistic clinical scenarios in a ‘safe’ environment [38]. In recent decades, a greater emphasis has been placed on active learning in small groups, with the uptake of problem-based learning (PBL) in most UK medical schools [39]. Moreover, simulation-based learning is becoming more commonplace in postgraduate medical education and is promoted by Health Education England for Core Medical Trainees [17] and the Intercollegiate Surgical Curriculum Programme for Core Surgical Trainees [18]. Whilst the medical students in this study supported the integration of simulated clinics within the undergraduate curriculum, they did not feel they could replace didactic teaching entirely. This was mostly related to concerns regarding the inability of simulated clinics to deliver large volumes of knowledge required to pass examinations. However, the students noted that this could be overcome with a prior didactic session, and that the two teaching modalities complimented one another.

### Knowledge gain

The medical students in this study felt significantly more confident after the simulated clinic in their ability to safely and effectively assess breast lumps, with greatest confidence gains in asking patients about their risk factors for breast cancer, performing and documenting a breast examination, and ordering the correct investigations. These domains require the application of knowledge assimilated from textbooks or classroom-based teaching to clinical practice. Simulated clinics therefore help to bridge the gap between acquiring knowledge during medical school and applying this in clinical practice.

### Strengths and limitations

The findings of this study should be interpreted in the context of its strengths and limitations. Students were asked to compare a didactic tutorial with the simulation session; however, the didactic session was always held first which may have confounded student opinion. The sample size was small (*n* = 17) and participants were recruited from one teaching hospital, limiting generalisability. However, the rich qualitative data collected provided a valid substrate for thematic analysis performed using rigorous published methodology by three independent coders. Moreover, the transcripts reached a point of data saturation, suggesting adequate exploration of the students’ views.

## Conclusion

This evaluative case study of surgical simulation clinics, based on a SECO clinic design, has demonstrated important motivations and preferences for learning amongst clinical medical students. In particular, the simulated clinics promoted a shift towards intrinsic academic motivation by allowing students to recognise the importance of preparing for clinical practice as opposed to focusing on written examinations. Surgical simulation clinics were received by the students as a positive addition to the undergraduate curriculum. Integration of surgical simulated clinics into the undergraduate curriculum could facilitate acquisition of clinical skills through active learning, a method highly valued by students. Further research is required to validate these findings in larger cohorts and other surgical and non-surgical teaching settings, and to examine the impact of this teaching on preparing medical students for the transition from medical student to clinician.

## Supplementary Information

**Additional file 1: Table S1.** Example of feedback form given to students based on patient-centred outcomes, filled in by the simulated ‘patient’. **Table S2.** Example of feedback form given to students based on clinical outcomes, filled in by the tutor facilitating the station. **Table S3.** Anonymised transcripts from the sessions held in November 2019 and January 2020. The students were debriefed in two groups, hence two transcripts per session.

## Data Availability

Anonymised raw questionnaire data and transcripts are available as supplementary information files.

## Abbreviation

SECO: Safe and Effective Clinical Outcomes
