# Supplementary material for: Whole consultation simulation in undergraduate surgical education: a breast clinic case study
Source: BMC Med Educ. 2021 May 28;21:305. doi: 10.1186/s12909-021-02757-x (PMC8164306; doi:10.1186/s12909-021-02757-x)
Supplement: Supplementary file 1 — Additional file 1: Table S1. Example of feedback form given to students based on patient-centred outcomes, filled in by the simulated ‘patient’. Table S2. Example of feedback form given to students based on clinical outcomes, filled in by the tutor facilitating the station. Table S3. Anonymised transcripts from the sessions held in November 2019 and January 2020. The students were debriefed in two groups, hence two transcripts per session. [file 12909_2021_2757_MOESM1_ESM.docx]

**Whole consultation simulation in undergraduate surgical education: A breast clinic case study**

Alice Lee^1^, Dalia Abdulhussein^1^, Mohammad Fallaha^1^, Olivia Buckeldee^2^, Rory Morrice^2^, Kathleen Leedham-Green^3^

**Author affiliations:**

1. Academic Foundation Doctor, Department of Surgery and Cancer, Imperial College London, London, SW7 2AZ, United Kingdom
2. Associate Clinical Teacher, Faculty of Medicine, Imperial College London, London, SW7 2AZ, United Kingdom
3. Medical Education Fellow, Medical Education Research Unit, Faculty of Medicine, Imperial College London, London, SW7 2AZ, United Kingdom

**S1.** Example of feedback form given to students based on patient-centred outcomes, filled in by the simulated ‘patient’.


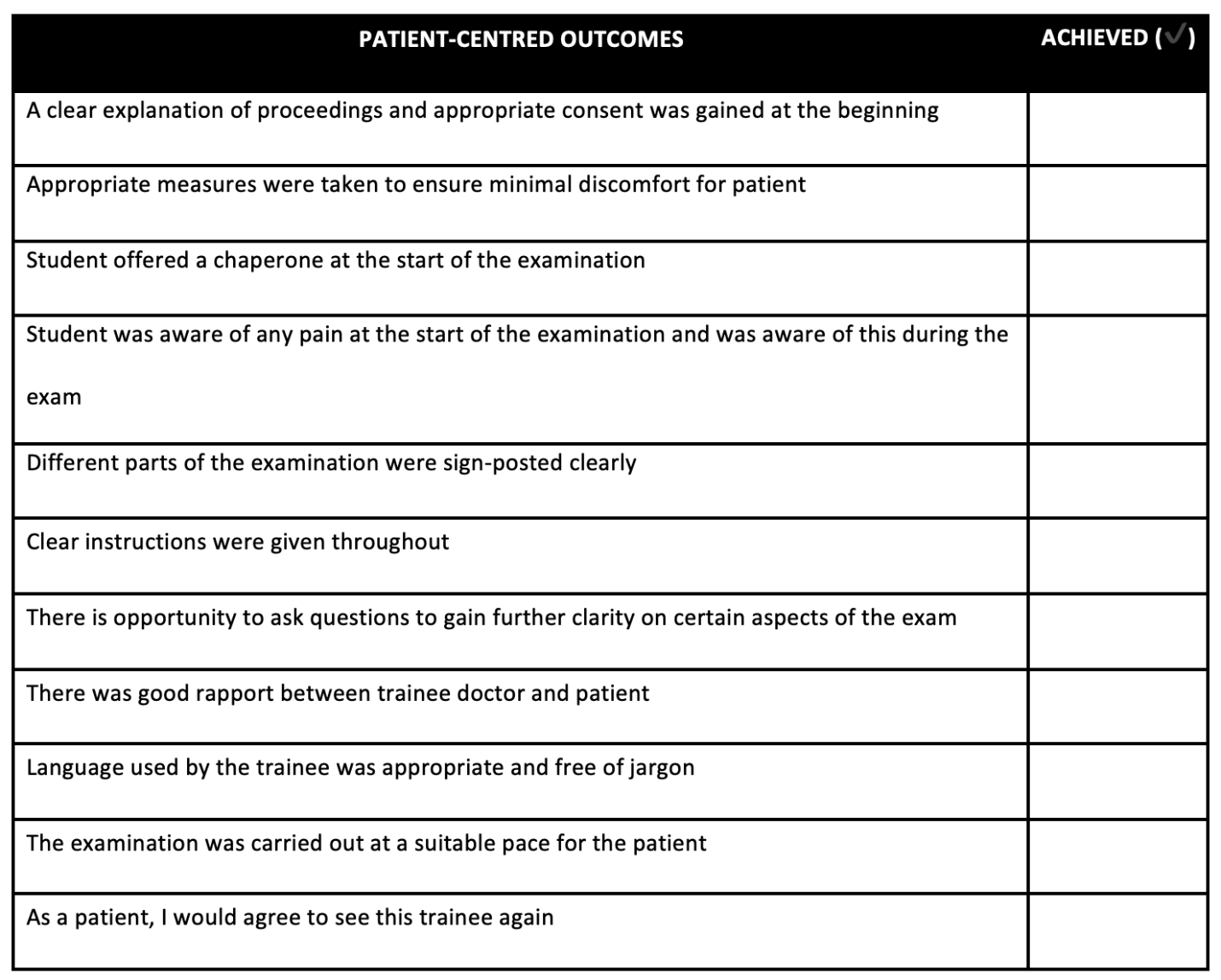


**S2.** Example of feedback form given to students based on clinical outcomes, filled in by the tutor facilitating the station.


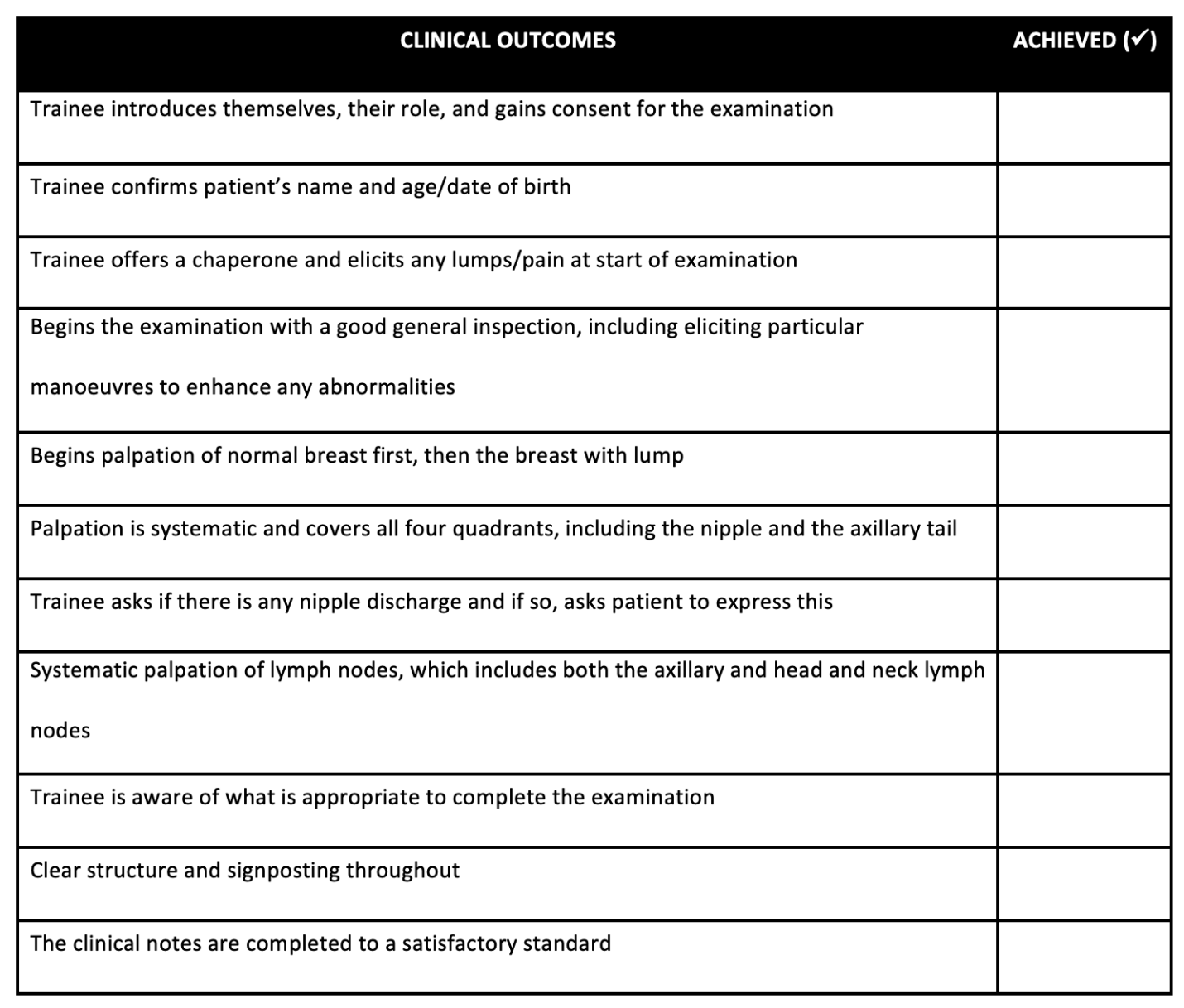


**S3.** Anonymised transcripts from the sessions held in November 2019 and January 2020. The students were debriefed in two groups, hence two transcripts per session.

**Debrief transcript 1 – November 2019**

Student 1: Okay, so guys, first question: how did you feel to learn in a simulated clinical environment?

Student 2: Sorry?

Student 1: How did you feel to learn in a simulated clinical environment?

Student 2: How did I feel about it?

Student 1: Yeah

Student 2: Erm .. I don’t know, I don’t really have any apprehensions, I was quite looking forward to it. It turned out to be quite nice. Yeah. Enjoyable.

Student 3: It was definitely different to the normal teaching that we received.

Student 4: Yeah I definitely agree. It’s more productive, you do more and the teaching fellow were quite nice and approachable.

Student 2: I think it’s the fact that it’s such a small group. It’s more personal and you can actually .. i don’t know … you learn more because … it’s more intimate, you can talk to people better and voice your concerns etc.

Student 1: Okay so this second question: this simulated virtual clinic was based on the same learning outcomes as the breast tutorial last Tuesday. How did you feel each session addressed these outcomes?

So the breast tutorial we had, we also went through the examinations, and at first it was just talk on the investigations and also the different diseases you can have. I found it quite useful then too, but with this it was more like, you were out there. I don’t know, it kind of replicated more of an OSCE kind of style...

Student 2: Yeah

Student 1: … than what we had before.

Student 2: Yeah

Student 3: That being said though, I don’t think I would have found this session as useful if I hadn’t done that one already

Student 1: Yeah I agree actually, because I kind of  took what I learnt from there to this one especially with the examination.

Student 2: I think it complemented what we had learnt really really really well, but as a standalone  thing, it probably wouldn’t have enough information on it. It didn’t really go into detail about the specifics of each disease as such, but as a complement to what we already learnt it was amazing.

Student 4: Yeah I agree actually

Student 3: Yeah you need a time [inaudible] or little break inbetween to process it. If we’d had it like, straight after eachother, it would just be overwhelming.

Student 2: Yeah, that.

Student 1: I mean, if we know from now on these sessions are going to go like this, and they tell us what the sessions are going to be on, if they tell us before, then maybe if you do your own pre-reading instead of having two different sessions …

Student 2: Yeah

Student 1: … and then come in knowing what to expect, then I guess it would be okay.

Student 3: Covering the content before these sessions would be good.

Student 1: Yeah. Okay so do you prefer to learn in a simulated clinical environment or a classroom based tutorial on the same subject matter?

Student 3: If it’s just one of them [session] then it would probably be the classroom.

Student 2: I don’t know, this builds your practical skills a lot better, and I feel like it gives you better information for like the history taking part. The history taking part was amazing, like I personally thought that the history taking part was so useful, but actually knowing specifically about the disease and all the different investigations and symptoms and everything that can come with it can only really be well taught in a classroom environment. Although to be fair we didn’t really have a station where we went through the different diseases and talked about them together so I think that to be fair it’s not really representative, we can’t really say, because it’s not like we sat down and spoke about all the different types of breast lumps you could have so I think that if we had that that could have been really useful … it could have been more useful than a classroom experience. But until we have that, we can’t really say.

Student 1: I agree. Okay: students presented with two printed statements. The top priority of my education should be to prepare me to pass final exams; the top priority of my education should be to prepare me to be a competent junior doctor. Discuss to what extent you agree or disagree with those statements

Student 2: Could you read those out again?

Student 1: So either your top priority is to prepare me to pass my final exams, or to prepare me to be a competent junior doctor.

Student 2: That’s a tricky one really, isn’t it. [Student name] how do you feel?

[PAUSE]

Student 1: So do you feel that your main priority is to prepare you to pass your final exams or to actually be a good, confident junior doctor?

Student 3: I think it’s a bit of both

Student 2: A bit of both, yeah.

Student 1: As medical students, especially when the deadlines are coming, I mean, passing exams is kind of our main priority. But at the end, we actually are going to be doctors so

Student 2: I think at this stage, our main priority if we’re being realistic about it is to pass our exams. We can be the most competitive junior doctor, but if we haven’t passed our exams, then you know [inaudible]... Yeah I think as you go along, and as you become better equipped for your exams, everyone has got it at the back of their mind “Oh I want to become a good doctor” but at the same time, I think it’s a bit of both, but I think exams are our top priority.

Student 3: I agree

Student 2: Okay

Student 1: Okay how do you feel simulated clinical sessions and classroom based tutorials prepare you for these educational priorities: passing exams versus competency as a junior doctor?

Student 2: Sorry, say that again

Student 1: So how do these clinical sessions, the one we just had now, versus the classroom based tutorial prepare you for your educational priorities, you know, passing exams, and competency as a junior doctor.

Student 4: I mean we have an OSCE at the end so of course these kind of practices are very useful to help us pass our exams

Student 2: I think simulated clinic sessions make use a lot more competent as a junior doctor, rather than focus on helping us pass exams. Actually to be fair, I think the simulated, they’re really useful for OSCEs, I mean they’re amazing for OSCEs. Yeah I really enjoyed the history taking aspect of it, and the practical taking an examination part. I don’t really get that … even when you go out on wards, it’s not really the right environment, I feel more at ease doing it in this environment than doing it on the ward. I feel like I learn better here than on the ward, doing exams and taking histories that is.

Student 4: Yeah I mean, I feel it kind of does prepare us for the exams, but also kind of tests our professionalism and maturity, and that kind of makes you … determines whether you are a competent junior doctor as well, so again, kind of both I guess.

[PAUSE]

Student 3: Yeah I agree.

[END]

**Debrief transcript 2 - November 2019**

1. How did it feel to learn in a simulated clinic environment?

It was interactive. I barely noticed time was passing.

Yeah same. I feel like it’s more appropriate to a real-life situation.

I wouldn’t teach about disease this way but as far as examination goes it probably makes sense to teach it this way.

Yeah that’s precisely it- for what we were learning it was highly suitable.

And also it’s better to get feedback early on rather than learning it and then not doing it for ages and then doing it again and not knowing it.

Yeah so like consolidation sessions if they did it weekly it would be a really nice way to learn examinations.

Interacting with patients in history, examinations and recommending stuff to patients was extremely useful.

Being made to think on your feet is useful as it means you remember it.

2. The simulated clinic was based on the same learning outcomes as the breast tutorial last week. How did you feel that each session addressed these outcomes?

Its like almost the same question. They both have their merits.

I think there’s pros and cons for both. In terms of learning things like risk factors I think the first session where there are slides is more useful as we get to see stuff.

Learning things like differentials, you probably get more of that in the tutorial but recognising them is an entirely different scenario.

I think what made this sessions today really useful for me personally was we already had that session then afterwards we came and consolidated that session a few days later with this. It tested our knowledge.

Yeah.

Yeah.

It will be useful when youre flagging in the exam and your trying to think back to your teaching and you can remember this session.

Yeah.

It was good that it was now and not directly after the session.

I feel like integrating both into the same… I think both are needed for teaching.

Yeah I would say one is better but we need both, like one at the beginning of the week and one at the end.

1. Do you prefer to learn in a simulated clinic environment or a classroom-based tutorial on the same subject matter?

That’s what we’ve just spoken about. So we said we don’t prefer one over the other. Learning presentations and risk factors and baseline knowledge is better in a classroom but we need to have a simulated clinic environment to tick off risk factors in our heads and be comfortable ruling things in and ruling things out.

It’s one thing to know what the symptoms are supposed to be and another thing to recognise them on a patient, even a simulated one.

And when you’re placed in front of a patient you have to process everything in your head and rule things in or out. We could easily list the symptoms and stuff but we couldn’t…

The second part of what we’ve done today is skills based. A lot of it is skills based like being able to speak to your patient properly and then understand what they’re saying.

Yeah, understanding your patient manner as well and like with sensitive things like breast cancer to learn how you’d approach it as well because you have to be more sensitive.

1. Students are presented with two statements: “The top priority in my education should be to prepare me to pass my final exams. The top priority in my education should be to prepare me to become a competent junior doctor”. Discuss to what extent you agree or disagree with those statements and why.

I know people who come to the bare minimum of everything and still pass their exams. You put in the work in your private time then most of the time passing the exams isn’t that hard but showing competency in a clinical environment- if you don’t come to firms then you won’t know what you’re doing in a hospital so probably the latter- 75:25 for me.

Prioritising final exams will eventually catch up to you.

To be honest prioritising my exams is probably my priority at the moment, if I’m honest. Sometimes I do some extra stuff like I can go and take another history if I’ve already done one so that I can learn to be a competent doctor later on but right now my priority is just to pass my exams and beyond that is kind of aside.

I was saying that last year one thing I took from this experience as opposed to just taking obs is that what I’m learning now what about when I’m going to have to recall that knowledge as a doctor. I have that awareness know that one day I might have to implement this knowledge as a doctor. But right now I do feel like I am still quite tailored to passing exams and remembering content for exams.

Yeah but I’ve had to take over from a final year before because he couldn’t remember how to take blood. This is the only clinical year we get for a long time so if we don’t get as much out of it as we can it will come back to bite you. To be honest focusing only on the academic stuff will impact you when it comes to the more clinical and practical stuff to be fair.

Yeah.

At this moment I’m probably like 25:75 good doctor just trying to learn good ways and mannerisms. Like I’m not saying that will be the case when it gets to third time, it will probably flip completely.

1. How do you feel simulated clinic sessions and classroom-based tutorials prepare you for these educational priorities (passing examinations vs. junior doctor competencies)?

I think personally it does prepare you more so than what we were taught before. Like before it was more passive learning but actually doing it yourself is something different and having feedback from seniors is really useful.

Without the pressure of doing it in real life things are much easier to recall and because we will be and have been in a similar place to what that person has been we can pick up on things they have done that we wouldn’t have done.

It was a very positive experience. The simulated sessions are useful for both competencies as a doctor as well as practical things like passing our OSCE. Classroom based tutorials are better for written exams and form a baseline knowledge for clinical ability but to pass written exams we need to be taught stuff in the classroom.

In reality you need both. Imagine we had the virtual surgery course matched topic to topic with the lunchtime tutorials we had it would be really good.

I think what was good about virtual surgery is that they did the teaching but they also made us do stuff like examinations too. For me this helps me remember stuff a bit more.

And also with history taking as well, practicing the extra questions you’d ask in an abdo history over and above your standard history makes it useful to remember.

It’s all about consolidation really.

A nice idea would be to have two sessions a week, the first as classroom based and then later on that week would be simulated clinical environment to consolidate that.

**Debrief transcript 1 - January 2020**

Faciliator: how did it feel to learn in a simulated clinic environment?

Student 1: I thought it was really good

Student 2: I felt ?more confident to ask questions and get things wrong because obviously it wasn’t a real patient so it was good to interact in this environment and use a model… this is the place to make mistakes rather than on the wards

Student 3: I think part of that was because it was a small group as well

Student: Yeah it’s just three people its really good

Student: And they explained it in a really straight forward way, we went through it very gradually which was good

Student: And it was very relevant to the stuff that we’ve been doing on the wards so you can apply it which was quite good

Student: I guess it’s also like lot more time for us to go through it because its very rare that you see a patient from presentation to future investigations and maybe we wouldn’t have had the chance to do that on the ward…

Facilitator: and when you were in it did it feel real? Did it feel more real than just a tutorial or something?

Student: yeah definitely, we had like, you felt what it was like to do it in real life. Obviously it’s a bit different because you’re doing it with your friends but still going through like sequence and actually thinking ‘what do I do next?’ is helpful i think

Student: it’s good to be put on the spot as well, because I think just a tutorial is quite passive and so you could be like ‘I could do that, I can do all these things’ but then when you actually go to do it in a simulated environment you’re like ‘oh wait hang on a minute’

Student: that’s the thing, they’re very good teachers, like they explain things in a very straightforward way because some people just can’t explain things whereas they can

Student: I feel like they are making us do the stuff, like it identifies what we actually do know and what we’ve retained and what we don’t

Facilitator: Great, ok. So, the simulated clinic that you did was based on the same learning outcomes as the breast tutorial that Liv did last week. Erm, so how do you feel that each session like addressed those outcomes for you and the learning that you got from it?

Student: i felt like both of them were useful in their own way, and in fact I felt doing this after the tutorial was actually better because it consolidated all of the stuff that we did in the tutorial so I think they both kind of go hand in hand which is kind of a good thing, but maybe just look at it as an adjuvant rather than one or the other.

Student: hmm yeah, I think they were both very good because i feel like the first one was more like our actual learning outcomes like our conditions that we need to know and to have a clear image of what the differentials could be and then the second one was like how would you use all this knowledge in practice

Student: And I mean that’s kind of how imperial have done the year, so like we have the first two years as preclinical because you actually need that foundation of knowledge before you go into like the clinical environment and start applying it, like its very difficult to go into things completely blind, like not knowing anything

Student: I think the first tutorial prepares you for the writtens and this one prepares you for your OSCE

Student: yeah, exactly

Facilitator: Ok, erm, and do you feel like if you were only to have one or the other would there be one that you would-

Student: I would want both

Student: I would want both, but if I could only have one I’d select this because the book stuff you can just look it up on your own time whereas you can’t recreate this by yourself

Student: Yeah. I would say if you were to replace tutorials with this, it would be good to give the topic beforehand…

Student: yes…

Student: …so that you could maybe like look at the theories so that you can then apply them in the practical aspect, I think having a tutorial like a week ago not knowing what the topic is today, is probably the best thing because you learn some stuff in the tutorial, you forget about it, and then you have to use active recall to remember the info, whereas if you knew the topic just before the session you’re just gonna cram it, you’re not gonna see what you remember, and I feel like the most useful part of this was, for example, the history, when we were doing the history taking I remembered some of the risk factors from the tutorial and then the ones that I forgot I knew I had forgot so now when I go to do an actual history I’ll make more of a conscious effort to ask the stuff that I’d forgotten.

Facilitator: hmm. And which do you feel like you prefer, which is more enjoyable for you do you think?

Students (multiple): this one, for sure

Student: It’s a lot more engaging when it’s like in small groups, and you’re supervised, you can’t just like zone out

Student: And you can also ask questions and like discuss things

Student: I think because there’s only 3 people you just cant zone out because its gonna get picked up, whereas when there’s 20 people if you sit at the back you don’t have to do anything

Facilitator: Right, OK. So I’m going to read two statements the first one is ‘the top priority for my education should be to prepare me to pass my final exams’ and the second one is ‘ the top priority for my education should prepare me to be a competent junior doctor’. So to what extent do you think you agree or disagree with each statement and why.

Student: I think the second one, I mean I agree the most with because if you’re gonna be a competent junior doctor hopefully you’ll pass your final exams like that will come with it as a given but then you’ll be more used to what the life will actually be like then, whereas if you just learn theory then I feel like you will be less prepared

Student: I feel like in the clinical setting you like pick up things that I don’t know if you can really learn from an exam like all this information that F1s tell you just in general, and then you see like the same things come up again and again and you realise like what’s important, what do you actually have to do, you can’t get that from a book I don’t think

Student: I feel like when you graduate the job of a junior doctor is very different, like its mainly admin stuff and I feel like you need to have an element of passing your finals because you’re not gonna make it to being a junior doctor if you don’t pass your finals.

Student: I think like in terms of having time to go through the knowledge, you probably won’t get that when you graduate kinda thing, because I think a lot of the junior doctors say like you probably know the most once you’ve passed your finals so you do need to ?know your stuff (06:59)

Student: I feel like this might be a bit controversial but uhm most of the learning happens when you qualify, after you’ve qualified, so its not really like feasible to aim to be an amazing doctor by the time you leave medical school so yeah… just thought I’d add that

Student: but also at the same time like, apparently when you graduate, its like a huge change, youre literally just thrown to the wolves, so if you don’t put the work in at medical school and are expected to like be able to do certain things on your first day as an F1, its like you need to prepare yourself for that

Facilitator: Do you think that your priorities might change as you progress through medical school?

Student: I think inevitably, like, its like well yeah whatever situation youre in at the time, that’s gonna be your mindset when you’re answering this question. So i think if you asked us like right you know a couple of weeks before the exam we’d be like yes med school like (muffled)

Students (multiple): yeah, yeah

Student: At the start of this year I was like, ahh lets really get a feel of what it’s like on the wards, whereas now, I’m already more like ahhhh give me ???? (0815) for the exam blab la bla whereas at the start of the year I wasn’t even thinking about that, so even within a year it changes quite a bit

Student: I feel like when you get to sixth year its- finals are approaching but then youre also like oh a couple months after that i’m gonna be the F1 and im gonna be doing nights and covering all the wards, I feel like its, I dunno

Student: Its different pressures at different stages isn’t it

Student: Yeah, I feel like we’ll probably be stressing about both in sixth year

Student: Yeah, but we’ve got time until then!

Facilitator: So how do you feel that simulated clinics with patients like this and classroom-based tutorials prepare you for these differing priorities so passing exams and being a competent junior doctor.

Student: i think we’ve already mentioned like the tutorials are more like more for exams and the tutorials are more for OSCES. I think what helps with tutorials is like the case presentations and that’s like a little bit different to some of the lectures we’ve done so that also like play into the OSCEs and to like clinical practice… I think you need both the tutorials and simulated clinics to do well

Student: I think the simulated clinic, its pretty much what they do in the breast clinic, so its like very much what we’ll have to do as a doctor, so I feel like in terms of that respect, this is more useful than a normal tutorial

Student: Yeah and also, like having different specialties like for all the clinics, because I don’t think well have the chance to sit in on all the clinics so this would be the place to do it to like learn about things that we haven’t been able to see

Student: I think particularly for the niche-er exams as well, because like respiratory, cardio, abdo, neuro, we’re gonna get lots of opportunity to do that, but these sort of niche-er ones I think its good to get

Student: yeah because we’re not all gonna do breast

Student: yeah it totally depends which firm you’re on so its good to get exposure to all of them

Student: then its more like standardised, the teaching, because it does feel like sometimes, I don’t know, like, you’re worried you’re missing things or that…

Student: …you could be disadvantaged depending on where you are…

Student: Yeah, so its good to like, have standardised teaching, rather than just (muffled, 11:01)

Student: I’d say as well in like textbooks and stuff, they’ll have like a billion investigations, so you don’t necessarily know which one is the one that you’ll use first in the hospital, whereas by doing simulated clinics you’ll see oh okay this is the first line, this is what you progress to because its got better specificity etc etc

Student: …like the point of the session as well was when to ask for help and like how to do that and there’s no other way really to learn about it in a textbook kinda thing

Student: ….and then yeah when is it appropriate to ask someone what to do, because obviously you’ll find yourself in the first situation maybe you’re panicking

**Debrief transcript 2 - January 2020**

1. How did it feel to learn in a simulated clinic environment?

I thought it was really good. I don’t think we’ve had the opportunity to do anything like this before

Having the opportunity to go through each of the stages as well was really useful to actually practice it.

It was good to be put on the spot too. Instead of a nice comfortable environment you’re just put on the spot which is more like real life and our actual osce exams – that was really useful.

1. The simulated clinic was based on the same learning outcomes as the breast tutorial last week. How did you feel that each session addressed these outcomes?

I think having a tutorial is a good pre-session for this.

Yeah I agree.

To take a specific or focused history you need to know about menopause and menarche which we wouldn’t have known without a tutorial.

Q: How do you feel when you’re sat in that sort of tutorial vs when you’re made to do something practical?

I thought this was good because if you’re put on the spot and donm’t know the answer then the tutor can go over it again with you and it becomes easier to recall it again in the future. I said the wrong thing before and now I’ll remember it.

I think because we’re in small groups in this session it makes it easier to get quick feedback compared to in a larger tutorial. In a big group you’re less likely to put your hand up to answer a question. But then if you do get it wrong in this setting then you’ve got a smaller group where you’re not disturbing too many people if you get it wrong.

Q: If you have a lunchtime tutorial, do you feel that you’re able to learn a lot from it or do you feel that you’re more likely to learn from this sort of session?

I learn more from practical sessions. In lunchtime tutorials there is just a lot of information so sometimes it just feels too much and it is hard to remember stuff until you actually put it to use and do it.

I personally feel that the structure of the breast tutorial was quite similar to this session but the majority of tutorials there is loads of information and loads of conditions so with those ones it would be nice split it up a bit more.

We got roughly the same information out of each session (tutorials and clinic) but it was just a different way of doing it.

Q: do you ever feel as if these simulated sessions are more exposing or make you feel more stressed?

Initially yes, but then I realized that if I don’t know or get it wrong then you can just ask your senior.

I didn’t feel stressed at all. I mean if you’re a doctor and your facilitator or senior is really nice then you’ll just be at ease.

1. Do you prefer to learn in a simulated clinic environment or a classroom-based tutorial on the same subject matter?

For this topic I wouldn’t have minded either. If we’d have just had the tutorial or this then I would have been fine but I feel like some other topics which are really information heavy then it would also be useful to give it a practical go.

Both together at once, like in an hour session we could do 15 mins of lecture followed by practical application.

1. Students are presented with two statements: “The top priority in my education should be to prepare me to pass my final exams. The top priority in my education should be to prepare me to become a competent junior doctor”. Discuss to what extent you agree or disagree with those statements and why.

I feel like I should agree more with the second one but realistically I just need to focus on my exams

I definitely don’t think passing your exams means you’re a good doctor at all.

No definitely not.

You need a patient manner and be able to communicate. Just knowing stuff alone isn’t useful. You need to be able to apply it and have techniques to make patients feel comfortable. Just knowing the principles of history taking doesn’t actually help you that much when you come to taking a history because if you can’t make a patient feel comfortable they won’t open up to you or talk to you.

Q: in year 3, do you think much about being a competent doctor or more thin king about exams?

I would say I focus more on how to be a good doctor because in hospital I’m always looking around at what other doctors are doing and learning from them, like ‘that was good’ or ‘no I wouldn’t do that’ and then this will help in the exams anyway especially with patients in our OSCEs.

I guess only when you’re in a clinical environment can you look and learn from other doctors and take their ways of how to approach and learn from certain situations.

Q: which would session would help you best to pass your exams vs. learn to be a doctor or is there no difference?

I think both sessions are necessary for exams and being a doctor.

If all our teaching was done in a simulated environment then I don’t think we’d feel as prepared to pass our exams. I think we would definitely still want some tutorials, like a balance is useful rather than all of one.

I think it depends on the topic. Like the breast clinic session is useful because we need to know the practical elements whereas some tutorials don’t have a practical element like IHD how would we do a clinic session for that.

Yeah it’s much easier for us to just be taught certain topics.

Well actually I think for all topics it would still be good to have a balance but for less practical topics we just skew the balance to having more of the session taught like 30-40mins taught then only 20mins practical.
